# The "Mobilisation" of the Medical Profession

**Published:** 1917-01-27

**Authors:** 


### THE "MOBILISATION" OF THE MEDICAL PROFESSION.

### (From the National Medical Union.)

In view of the somwhat alarmist and, as they think, misleading statements appearing in the Press on the above subject the Council of the National Medical Union at their meeting held on Saturday, January 13, considered the alleged difficulty in meeting the medical needs of the country, both civil and military.

The term "mobilisation" in the sense of the suggestions so far made appears to be synonymous with the conscription of a particular body of citizens who are more than warned that any lack of spontaneous alacrity on their part in offering their services for disposal according to the wisdom of a certain Central Medical War Committee will be followed by compulsory legislation, and the ousting of native by imported foreign medical service so far as the civil population is concerned.

Considering the whole-hearted response to the country's need which has characterised the medical profession in this crisis, a response which has frequently entailed much personal sacrifice, this attitude towards it by the powers that be seems neither just nor wise, nor has it, in the opinion of the Council of the National Medical Union, the justification even of necessity, if a proper use be made of the medical material available.

This Union, whose opposition to the Medical Benefit Section of the National Insurance Act is well known, has no desire to snatch any advantage for its views, but it expects a like self-abnegation on the part of those interested in the maintenance of the system of Medical Benefit referred to.

The Council of the National Medical Union, after full discussion and after consultation with cognate bodies in other parts of the kingdom, believes that every medical need, civil and military, may be met on the lines and upon the conditions hereunder indicated, which should be operative during the war and for a reasonable period thereafter until the situation is again tending to become normal and the abstract questions at issue in the medical world can again be considered. They believe also that medical services secured on these conditions will be willing services, a paramount consideration in the exercise of a profession in which physical ease has largely to be sacrificed and mental activity with much responsibility displayed.

The conditions indicated to secure such services are as follows:—

1. A primary condition to be repeal of the Medical Benefit Section of the Insurance Acts, thereby releasing many doctors who are unevenly distributed at present and releasing a population that would automatically distribute itself in the district.

2. Non-panel practitioners offering to attend the patients of panel doctors not to be required to attend them except as private patients.

3. Importation of English-speaking foreign doctors to attend the civil population is totally unnecessary and not in the national interests.

4. Compulsion in the case of the medical profession will only lead to disaster, and any voluntary organisation should be carried out by a properly constituted Medical Committee elected by and representative of the profession.

